# Patulin alters alpha-adrenergic receptor signalling and induces epigenetic modifications in the kidneys of C57BL/6 mice

**DOI:** 10.1007/s00204-024-03728-z

**Published:** 2024-05-28

**Authors:** Makabongwe Mazibuko, Terisha Ghazi, Anil Chuturgoon

**Affiliations:** Discipline of Medical Biochemistry, School of Laboratory Medicine and Medical Sciences, College of Health Sciences, Howard College Campus, University of KwaZulu-Natal, Durban, 4041 South Africa

**Keywords:** Patulin, α-1 Adrenergic receptors, DNA methylation, Extracellular signal-regulated kinases, Mitogen-activated protein kinases, Phosphatidylinositide-3-kinase

## Abstract

Patulin (PAT) is a food-borne mycotoxin produced by *Penicillium* and *Byssochlamys* species. It is widely known for its mutagenic, carcinogenic, and genotoxic effects and has been associated with kidney injury; however, the mechanism of toxicity remains unclear. To address this gap, we conducted a study to explore the changes in α-adrenergic receptor signalling pathways and epigenetic modifications induced by PAT in the kidneys of C57BL/6 mice during acute (1 day) and prolonged (10 days) exposure. The mice (20–22 g) were orally administered PAT (2.5 mg/kg; at 1 and 10 days), and post-treatment, the kidneys were harvested, homogenised and extracted for RNA, DNA, and protein. The relative gene expression of the α-adrenergic receptors (*ADRA1*, *ADRA2A*, *ADRA2B*) and associated signalling pathways (*MAPK*, *MAPK14*, *ERK*, *PI3K*, and *AKT*) was assessed by qPCR. The protein expression of ERK1/2 and MAPK was determined by western blot. The impact of PAT on DNA methylation was evaluated by quantifying global DNA methylation; qPCR was used to determine gene expression levels of DNA methyltransferases (*DNMT1*, *DNMT3A*, and *DNMT3B*) and demethylase (*MBD2*). PAT downregulated the expression of *ADRA1*, *ADRA2A*, *ADRA2B*, *PI3K*, and *AKT* and upregulated ERK1/2 and MAPK protein expression. Furthermore, PAT induced alterations in DNA methylation patterns by upregulating *DNMT1* and *MBD2* expressions and downregulating *DNMT3A* and *DNMT3B* expressions, resulting in global DNA hypomethylation. In conclusion, PAT disrupts α-1 and α-2 adrenergic receptor signalling pathways and induces epigenetic modifications, that can lead to kidney injury.

**Supplementary Information:**

The online version contains supplementary material available at 10.1007/s00204-024-03728-z.

## Introduction

In recent years, there has been an alarming increase in mycotoxin contamination, leading to a significant decline in the yield and quality of agricultural produce (Mahato et al. 2021; Zhong et al. 2018). Approximately 25% of the food output worldwide is contaminated by mycotoxins (Aasa et al. 2022), necessitating the continuous demand to ensure the protection of human and animal health by combating mycotoxin exposure on a global scale. Patulin (PAT) is one of the most commonly isolated mycotoxins in apples and apple products (McKinley and Carlton 1980). It is produced by several species of *Byssochlamys*, *Aspergillus*, and *Penicillium* during food spoilage as a result of poor handling and storage practices (Raistrick 1943). Several studies have shown PAT’s mutagenic, carcinogenic, and teratogenic properties (Puel et al. 2010; Ramalingam et al. 2019), with evidence indicating that its exposure causes oxidative DNA damage (Pal et al. 2017), even at relatively low concentrations (Melo et al. 2012).

In response to the risks posed by PAT consumption to human health, the Codex Alimentarius Commission (CAC) established the maximum permissible level for PAT in apple and apple products at 50 µg/l (CODEX STAN 193-1995), and the Joint Expert Committee on Food Additives (JECFA) set a daily intake limit of 0.4 µg/kg body weight per day (Hussain et al. 2020; Shephard et al. 2010). However, despite these standard guidelines on permissible levels of PAT (Ioi et al. 2017), long-term ingestion of PAT-contaminated products often leads to several negative health outcomes (Pal et al. 2017). Acute and prolonged exposure to PAT causes vomiting, diarrhoea, gastrointestinal issues (including gastric ulcers and intestinal haemorrhages), immune system suppression, and kidney damage (Mahato et al. 2021; Puel et al. 2010).

Growing research provides substantial evidence of PAT’s nephrotoxic potential, given the kidney’s susceptibility to toxic substances as one of the main retention sites for the mycotoxin (Hou et al. 2022). Kidneys play an essential role in maintaining fluid and electrolyte balance, homeostasis, and hormone regulation (Hering et al. 2020). These organs are also responsible for the excretion of toxins and metabolic wastes, making them vulnerable to toxic insults (Kellum et al. 2021). PAT exposure can lead to kidney injury; however, the mechanism of action remains unclear. Therefore, this study contributes to this knowledge gap by building on the premise that PAT exerts toxicity through the disruption of signalling pathways resulting in oxidative stress, mitochondrial dysfunction, and cell death in major organs (Glaser and Stopper 2012; Zhou et al. 2010).

A recent study found PAT induced oxidative DNA damage in HEK293 kidney cells, leading to renal injury by targeting the α-adrenergic receptors expressed in the kidneys (Hering et al. 2020; Pillay et al. 2020). These receptors are activated by endogenous catecholamines (Michelotti et al. 2007) and engage in signal transduction through the extracellular signal-regulated kinases/mitogen-activated protein kinases (ERK/MAPK) and phosphatidylinositide-3-kinase/protein kinase B (PI3K/AKT) signalling pathways (Karkoulias et al. 2006).

Catecholamines such as epinephrine and norepinephrine activate α_1_‐adrenergic receptors, initiating a signalling cascade through the Gq/11 protein family and phospholipase C (PLC), which ultimately leads to the cleavage of phosphatidylinositol 4,5-biphosphate (PIP_2_) into diacylglycerol (DAG) and inositol triphosphate (IP_3_), releasing stored calcium (Ca^2+^) (Cotecchia 2010; Dorn et al. 1997). This results in the phosphorylation of key proteins essential for cellular functions, namely p21^Ras^, PI3K, MAPK, and ERK (Dorn et al. 1997; Alblas et al. 1993). Moreover, both α_1_ and α_2_-adrenergic receptors have been conclusively shown to enhance the hydrolysis of PIP_2_ to PIP_3_, facilitating AKT activation by phosphoinositol-dependent kinase-1 (PDK1) (Wang et al. 2017; Hein 2006). Upon activation, AKT initiates the phosphorylation of transcription factors and proteins, promoting cell growth, DNA repair, and apoptosis inhibition (Aksamitiene et al. 2012). Similar to the ERK/MAPK pathway, suppression of the PI3K/AKT pathway also culminates in apoptosis-induced cell death (Belcheva and Coscia 2002).

Epigenetic modifications influence gene expression without altering the DNA sequence in cells (Lu et al. 2020). These processes, such as DNA methylation, can profoundly impact gene expression patterns and cellular functions and have been linked to a wide range of diseases. DNA methylation has been associated with adverse health effects. It primarily occurs at cytosine residues within CpG dinucleotides, facilitated by DNA methyltransferases (DNMT1, DNMT3A, and DNMT3B) and methyl-CpG-binding domain family proteins (MBD2) (Jin et al. 2011). Recent studies showed that mycotoxins induced epigenetic modifications (Ghazi et al. 2019; Sugiyama et al. 2021); therefore, this study explores the underlying mechanism by which PAT induces these modifications, with a specific focus on DNA methylation. Understanding how PAT may contribute to diseases by disrupting this process represents a significant frontier in mycotoxin research.

While substantial evidence exists on PAT-induced molecular and cellular changes leading to adverse health implications for kidneys, further research is needed to understand the impact of PAT on epigenetic modifications, particularly DNA methylation. Similarly, the interplay between adrenergic receptor signalling pathways and renal function remains to be fully explored. Hence, this study aimed to investigate the acute (1 day) and prolonged (10 days) effects of PAT exposure on α-adrenergic receptor signalling (PI3K/AKT and ERK/MAPK pathways) and DNA methylation (measuring transcript levels of *DNMT1*, *DNMT3A*, *DNMT3B*, and *MBD2*) in kidneys of C57BL/6 mice.

## Materials and methods

### Materials

PAT (P1639) was purchased from Sigma-Aldrich (Missouri, USA). The DNA Methylation Quantification Kit (ab117128) was obtained from Abcam (Cambridge, UK). Western blotting equipment and reagents were acquired from Bio-Rad (Hercules, California, USA), and the antibodies were obtained from Santa Cruz Biotechnology (Dallas, Texas, USA) and Cell Signalling Technology (Danvers, Massachusetts, USA). Primer sequences were purchased from Inqaba Biotechnical Industries (Pty) Ltd (Pretoria, South Africa). All other reagents were sourced from Merck (Darmstadt, Germany) unless specified otherwise.

### Animal treatment

All experimental procedures were carried out in accordance with the recommendations of the University of KwaZulu-Natal Animal Research Ethics Committee (Ethics number: AREC/079/016) and the ARRIVE guidelines. Twenty C57BL/6 mice (male, 20–22 g each) were housed (temperature: 25 °C, humidity: 40–60%, 12-h light and dark cycles) at the Africa Health Research Institute (AHRI) of the University of KwaZulu-Natal, Durban, South Africa. The mice of 6 to 8 weeks were separated into two experimental groups at random (*n* = 10): the control group and the PAT-treated group. The control group received no treatment, whereas the PAT group received a daily dose of 2.5 mg/kg of PAT (prepared in 0.1 M of phosphate-buffered saline) using a syringe at a rate of 0.250 ml/23 g (Song et al. 2014; Jin et al. 2016) for a duration of 1 day and 10 days. The dose of PAT received by the animals was selected based on prior studies indicating antioxidant activity and was administered in the early mornings with consistency (Song et al. 2014; Jin et al. 2016; Xu et al. 2007; Liu et al. 2008). Five mice from each group were sacrificed by cardiac puncture at 1 day and 10 days post-treatment under halothane anaesthesia; all efforts were factored in to ensure minimized animal suffering. The kidneys were then harvested and stored (− 80 °C) in Cytobuster™ Protein Extraction Reagent (Novagen, 71009) and QIAzol Reagent (Qiagen, 79306) for protein RNA, and DNA isolation, respectively.

### RNA isolation and quantification polymerase chain reaction (qPCR)

Mice kidneys were homogenized in 500 µl of QIAzol reagent, where total RNA was extracted as per the method described by Ghazi et al. (2019). Following isolation, the quantification of RNA was done using the Nanodrop spectrophotometer (ND-2000; Thermo-Fisher Scientific) and then standardized to 500 ng/µl. The quality of RNA was assessed based on the A260/A280 absorbance ratio.

Complementary DNA (cDNA) was synthesized with the Maxima H Minus First Strand cDNA Synthesis Kit (Thermo-Fisher Scientific, K1652) following instructions from the manufacturer’s protocol. The mRNA levels of *ADRA1A*, *ADRA2A*, *ADRA2B*, *MAPK14*, *MAPK*, *PI3K*, *AKT*, *DNMT1*, *DNMT3A*, *DNMT3B*, and *MBD2* were evaluated through the use of PowerUp™ SYBR™ Green Master Mix (Thermo-Fisher Scientific, A25742) as per the manufacturer’s protocol. The samples were loaded per well in triplicate and amplified by the CFX Real-Time PCR Detection System (Bio-Rad). Each gene was subjected to initial denaturation (95 °C, 8 min), followed by 40 cycles of denaturation (95 °C, 15 s), annealing (temperatures indicated in Supplementary Table S1, 30 s), and extension (72 °C; 40 s). *GAPDH* was included to normalize individual mRNA expression levels. Data were analyzed on the Bio-Rad CFX Manager™ Software version 3.1, and the changes in mRNA expression were determined through the comparative threshold cycle (Ct) method (Livak and Schmittgen 2001).

### DNA isolation and quantification of global DNA methylation

Genomic DNA was extracted from mice kidneys as previously described (Chomczynski 1993); samples were incubated in 150 µl of 100% ethanol (RT, 3 min) prior to centrifugation (2000×*g*, 4 °C, 5 min), and the resultant supernatant was discarded. The pellets were incubated in 500 µl of 0.1 M sodium citrate in 10% ethanol (pH 8.5) (RT, 30 min) and centrifuged (2000×*g*, 4 °C, 5 min); this was repeated twice before being re-suspended in 1000 µl of 75% ethanol and incubated (RT, 15 min). The pellets were centrifuged (2000×*g*, 4 °C, 5 min), air dried (RT, 5 min), re-suspended in 300 µl of 8 mM sodium hydroxide buffer, and centrifuged again (12,000×*g*, 4 °C, 10 min). The concentration of the DNA was measured using the Nanodrop spectrophotometer (ND-2000; Thermo-Fisher Scientific) and standardized to 500 ng/µl. DNA purity was determined through the A260/A280 absorbance ratio.

Global DNA methylation was quantified using the Methylated DNA Quantification Kit (Abcam, ab117128), following the manufacturer’s protocol. The absorbance was measured at 450 nm on a spectrophotometer (SPECTROstar nano spectrophotometer, BMG Labtech). The percentage of 5-methylcytosine (5-mC) in total DNA was determined using the supplied formula (Supplementary Material) and expressed as a relative fold-change in comparison to the control.

### Protein isolation and western blotting

Western blot analysis was performed to assess MAPK and ERK1/2 protein expression. Mice kidneys were homogenized in 500 µl Cytobuster™ Reagent supplemented with protease and phosphatase inhibitors and centrifuged (10,000×*g*, 10 min, 4 °C). Crude proteins were quantified using the bicinchoninic acid (BCA) assay and standardized to 1.5 mg/ml. The protein samples were then boiled (100 °C, 5 min) in Laemmli buffer (comprising distilled water, 0.5 M Tris–HCl (pH 6.8), 10% sodium dodecyl sulphate, glycerol, 5% β-mercaptoethanol, and 1% bromophenol blue).

Subsequently, sodium dodecyl sulphate polyacrylamide gel electrophoresis (SDS-PAGE) was performed for the separation of proteins (150 V, 90 min), which were then transferred onto nitrocellulose membranes using the Bio-Rad Trans-Blot^®^ Turbo Transfer System (25 V, 30 min). Thereafter, the membranes were blocked (RT, 1 h) with 5% BSA in Tris-buffered saline with Tween 20 (TTBS; 150 mM NaCl, 3 mM KCl, 25 mM Tris, 0.05% Tween 20, distilled water, pH 7.5). They were then incubated overnight (4 °C) with primary antibody: p44/42 MAPK (Cell Signalling Technology, #9102S; 1:1000) and ERK1/2 (Santa Cruz Biotechnology, sc-93; 1:200), washed in TTBS (RT, 10 min) five times before being incubated (RT, 1 h) with anti-rabbit (Cell Signalling Technology, #7074S; 1:5000) secondary antibody conjugated to horseradish peroxidase.

The membranes were washed five more times in TTBS (RT, 10 min) before they were visualized for specific protein bands using the Clarity™ Western ECL Substrate Kit (Bio-Rad, 170-5060). Images were captured on the ChemiDoc™ XRS+ Molecular Imaging System (Bio-Rad). Membranes were immersed in 32% hydrogen peroxide (37 °C, 30 min) before blocking with 5% BSA in TTBS and incubated (RT, 30 min) with the housekeeping protein β-actin (Sigma-Aldrich, A3854; 1:5000) for normalization against corresponding protein expression. To analyze the protein bands, densitometric analysis was conducted with the Bio-Rad Image Lab Software version 5.1, and data were presented as a fold-change in relative band density (RBD) to the control.

### Data analyses

Statistical analysis of all data was conducted using GraphPad Prism version 5.0 (San Diego, United States) through the unpaired t-test with Welch’s correction. Five (*n* = 5) or four (*n* = 4) mice kidneys were analyzed for PAT and control, respectively (specified in the graph captions). The data were presented as mean fold-change ± standard deviation (SD) unless specified otherwise. The results were considered statistically significant, where *p* < 0.05.

## Results

### PAT altered the α-adrenergic receptor expression

**Fig. 1:**
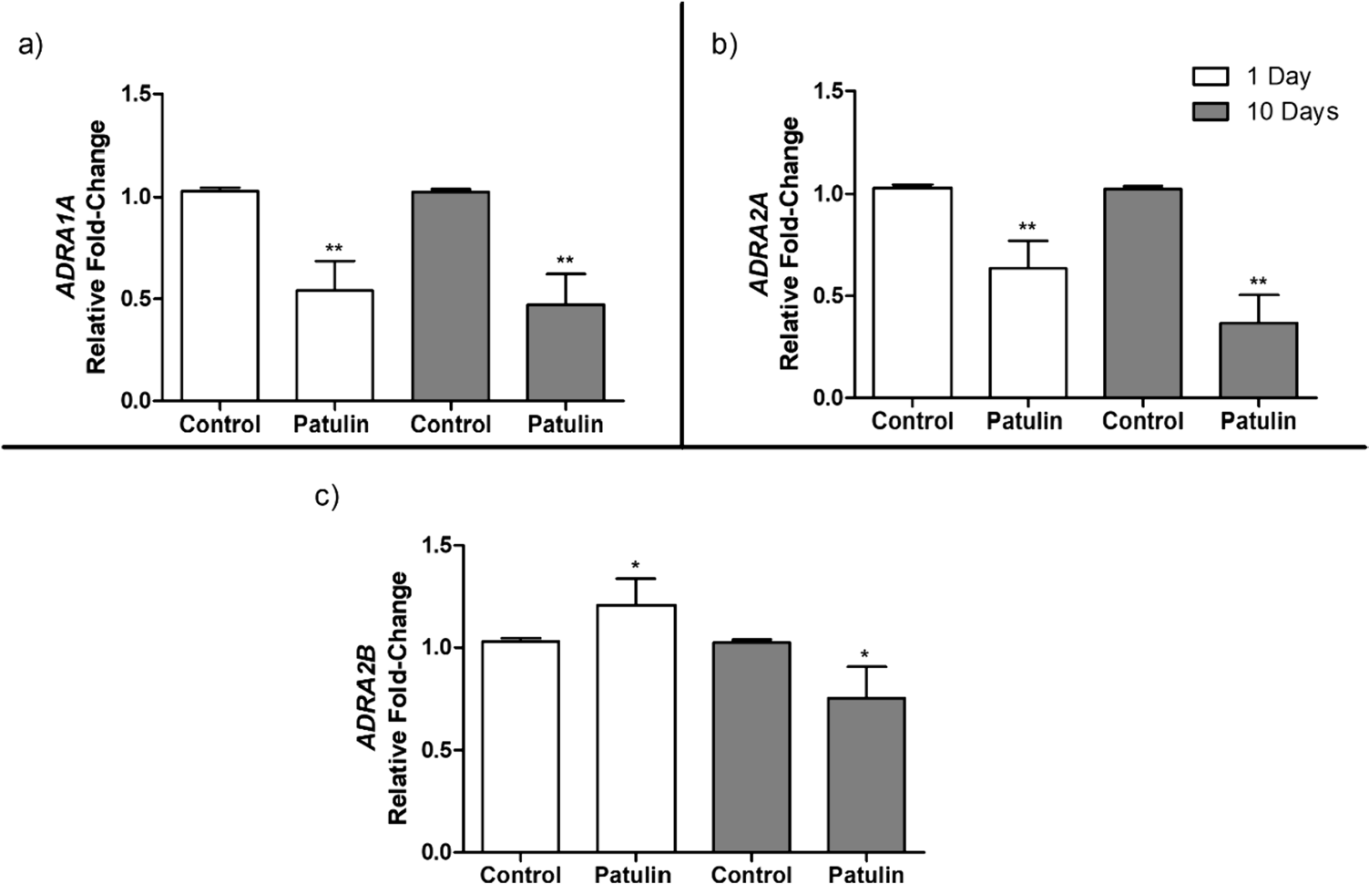
mRNA levels of **a** *ADRA1A*, **b** *ADRA2A*, and **c** *ADRA2B* in mice kidneys following acute (1 day) and prolonged (10 days) exposure to PAT. Data are presented as the mean relative fold-change (RFC) ± SD (*n* = 5). Statistical significance is denoted by asterisks (*p < 0.05; **p < 0.01) indicating differences between the PAT-exposed and control mice kidneys

PAT exposure significantly decreased (day 1) the mRNA levels of *ADRA1A* (*p* = 0.0018) and *ADRA2A* (*p* = 0.0029) in the mice kidneys compared to the control group (Fig. 1a, b), while there was a notable increase in *ADRA2B* mRNA levels (*p* = 0.0377; Fig. 1c).

Further, prolonged exposure (day 10) to PAT significantly decreased the mRNA expression of all three α-adrenergic receptors in kidneys relative to the control: *ADRA1A* (*p* = 0.0051; Fig. 1a), *ADRA2A* (*p* = 0.0024; Fig. 1b), and *ADRA2B* (*p* = 0.0357; Fig. 1c). These findings suggest that PAT negatively influences the expression of α-adrenergic receptors in mice kidneys.

### PAT upregulated the expression of ERK and MAPK in mice kidneys

**Fig. 2:**
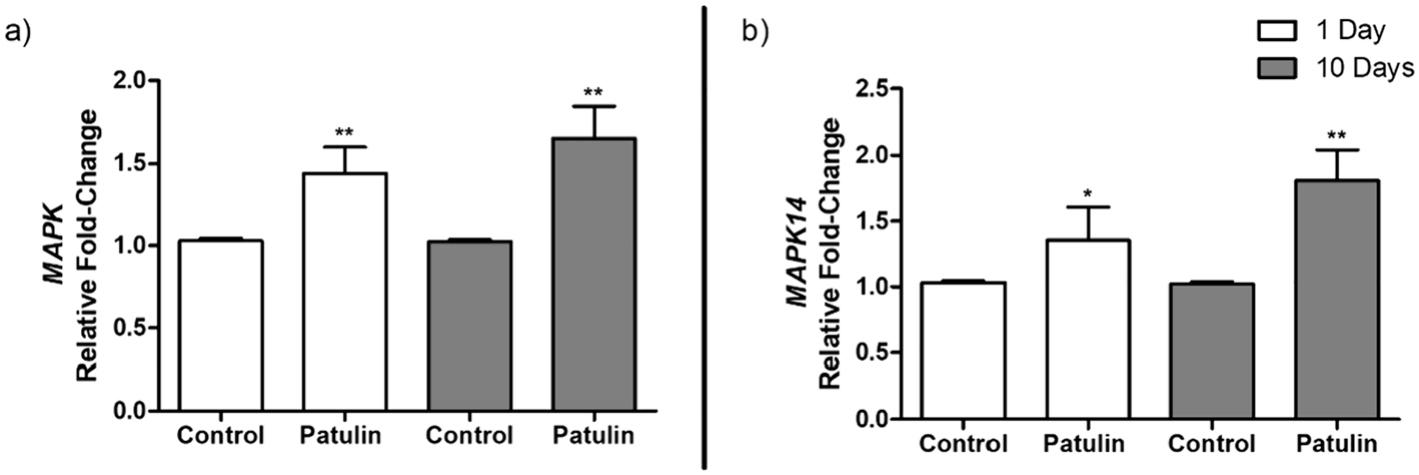
Gene expression levels of **a** *MAPK* and **b** *MAPK14* in mice kidneys exposed to PAT for acute (1 day) and prolonged (10 days) periods. Data are presented as mean RFC ± SD (*n* = 5). Statistical significance is denoted by asterisks (*p < 0.05; **p < 0.01) indicating differences between the PAT-exposed and control mice kidneys

The effects of PAT on the MAPK/ERK signalling pathway in the mice kidneys were assessed by measuring the transcript levels of *MAPK* and *MAPK14*. PAT significantly increased *MAPK* mRNA levels (day 1, *p* = 0.0048; 10 days, *p* = 0.0067; Fig. 2a). Similarly, *MAPK14* expression increased significantly (day 1, *p* = 0.0442; 10 days, *p* = 0.0068; Fig. 2b).

**Fig. 3:**
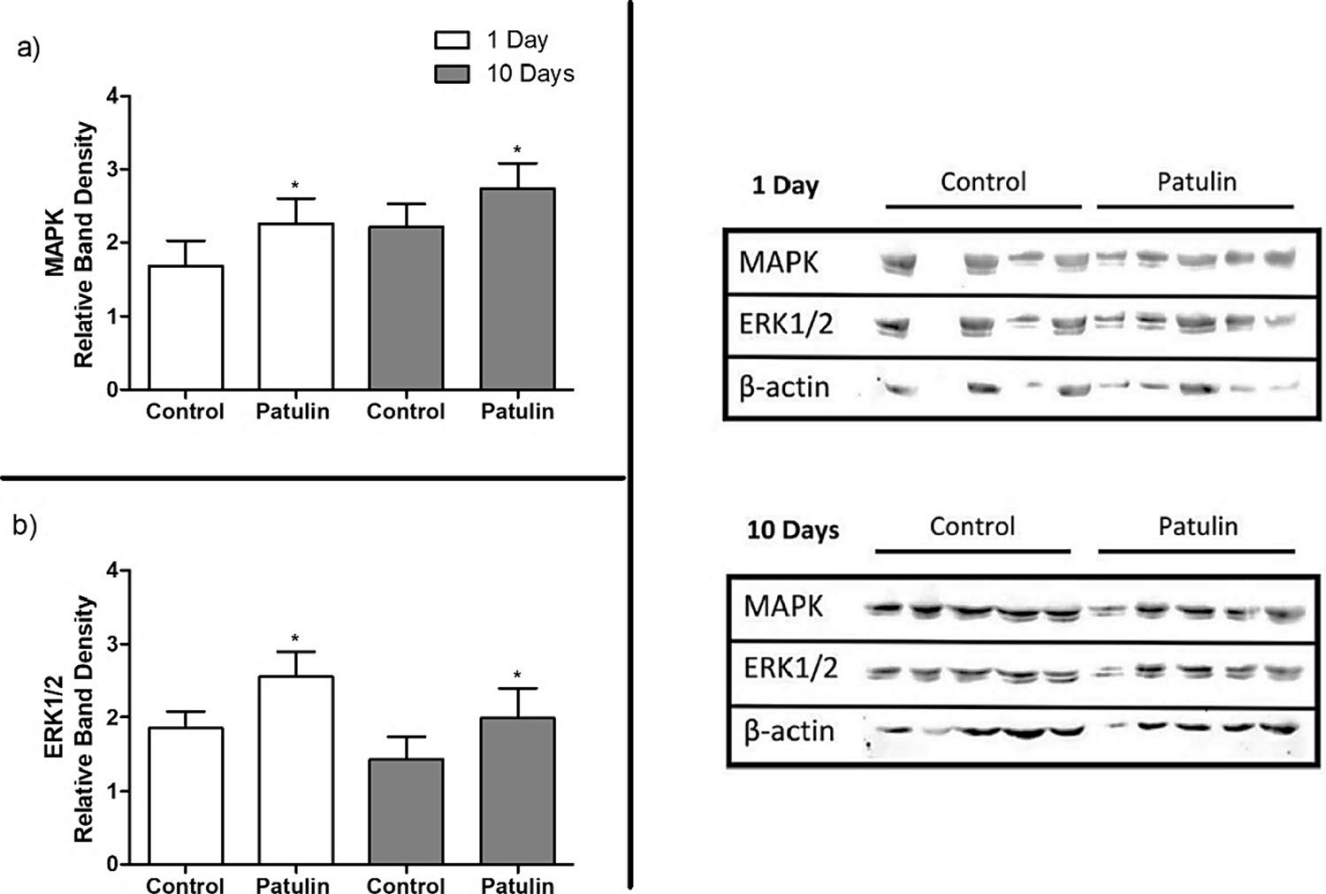
Protein expression of **a** MAPK and **b** ERK1/2 in mice kidneys following acute (1 day) and prolonged (10 days) exposure to PAT. Data are presented as the mean relative band density ± SD (*n* = 5, except for the control group at acute exposure where *n* = 4). Statistical significance is denoted by asterisks (*p < 0.05) indicating differences between the PAT-exposed and control mice kidneys

Western blot analysis revealed a significant increase in the protein expressions of both MAPK (day 1, *p* = 0.0464; Fig. 3a) and ERK1/2 (day 1, *p* = 0.0103; Fig. 3b) by PAT in the kidneys of mice. After 10 days of PAT exposure, significantly increased MAPK (*p* = 0.0404; Fig. 3a) and ERK1/2 (*p* = 0.0463; Fig. 3b) protein expressions as compared to controls. Thus PAT upregulates the MAPK/ERK signalling pathway, and potentially influences by the negative alterations of the α-adrenergic receptor.

### PAT altered the expression of *PI3K* and *AKT* in mice kidneys

**Fig. 4:**
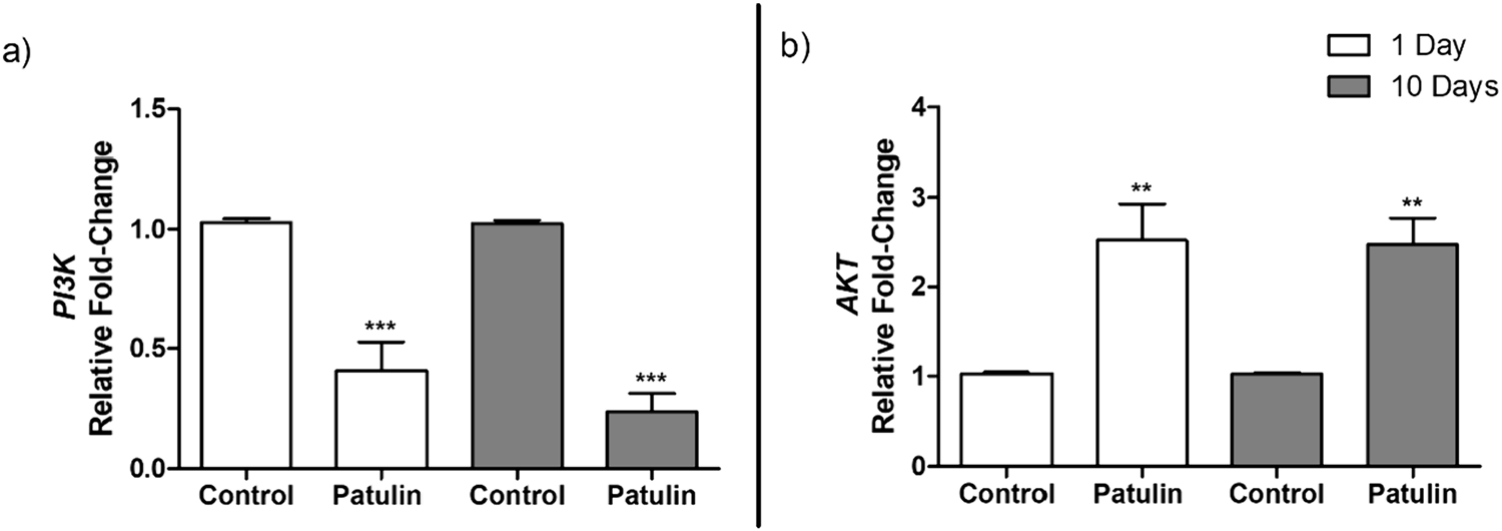
qPCR analyses of **a** *PI3K* and **b** *AKT* mRNA levels in mice kidneys following acute (1 day) and prolonged (10 days) exposure to PAT. Data are presented as the mean RFC ± SD (*n* = 5). Statistical significance is denoted by asterisks (*p < 0.05; ***p < 0.001) indicating differences between the PAT-exposed and control mice kidneys

To examine the impact of PAT on kidney function, we assessed the expression of *PI3K* and *AKT*; both proteins are involved in the regulation of various important cellular processes in the kidneys. PAT significantly decreased *PI3K* (day 1, *p* = 0.0003; and 10 days,* p* = 0.0002; Fig. 4a) and increased *AKT* expression (day 1, *p* = 0.0012; 10 days, *p* = 0.0023; Fig. 4b) in mice kidneys. PAT alteration of *PI3K* and *AKT* can lead to compromised kidney function.

### PAT-induced global DNA hypomethylation in mice kidneys

**Fig. 5:**
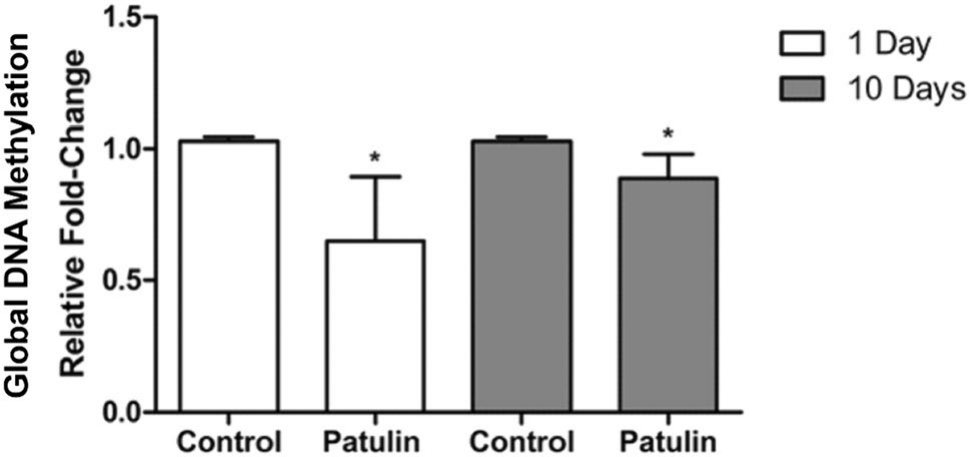
Global DNA methylation in mice kidneys following acute (1 day) and prolonged (10 days) exposure to PAT. Data are presented as the mean RFC ± SD (*n* = 5). Statistical significance is denoted by asterisks (*p < 0.05) indicating differences between the PAT-exposed and control mice kidneys

To understand gene regulation, the global DNA methylation status was measured. Quantification of 5-methylcytosine levels, a common marker of DNA methylation, revealed a significant decrease in global DNA methylation (day 1, *p* = 0. 0256; and 10 days, *p* = 0.0275; Fig. 5) in the kidneys of mice compared to their respective controls.

### PAT altered the expression of *DNMT1*, *DNMT3A*, *DNMT3B* and *MBD2* in mice kidneys

**Fig. 6:**
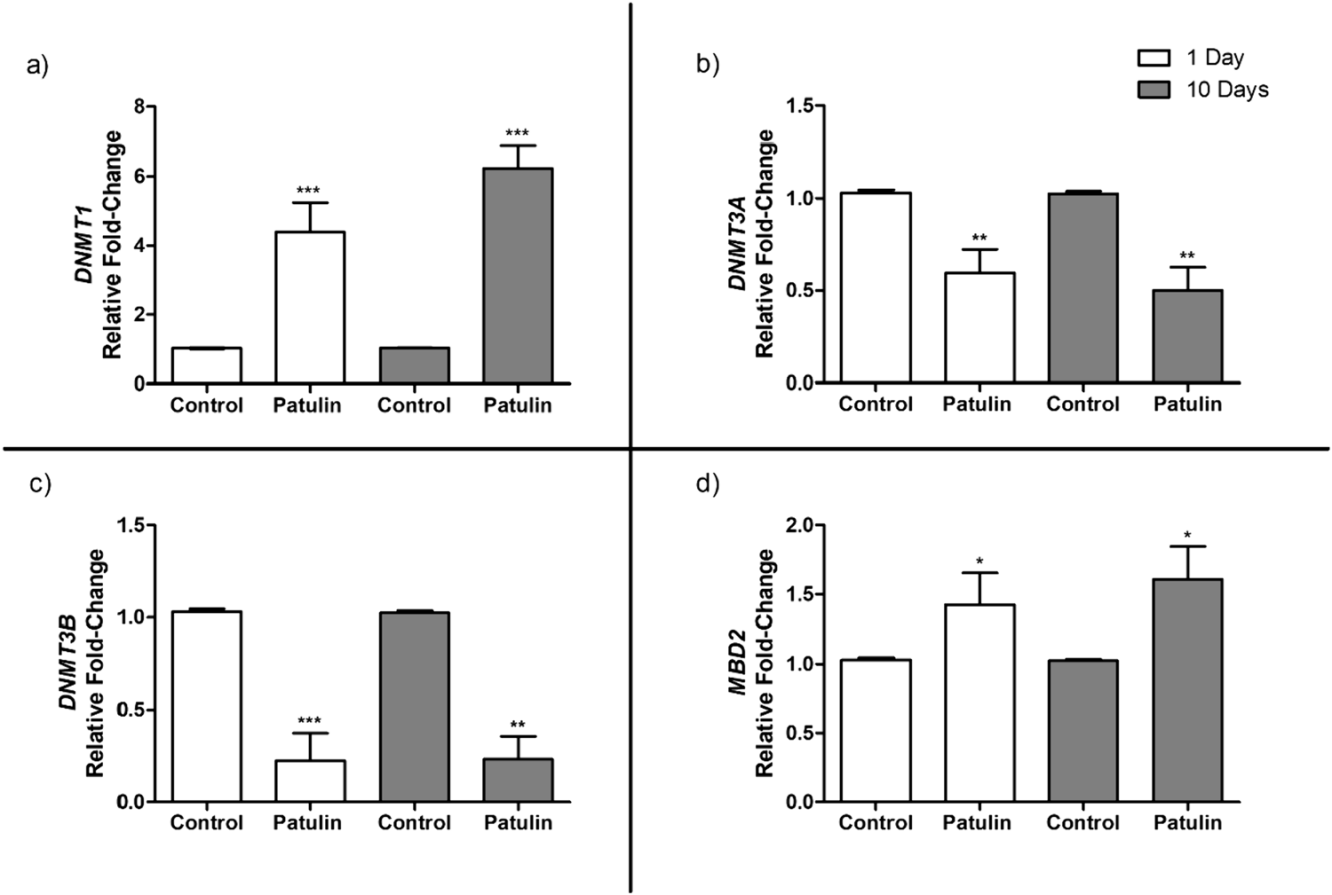
mRNA levels of **a** *DNMT1*, **b** *DNMT3A*, **c** *DNMT3B*, and **d** *MBD2* in mice kidneys exposed to PAT for acute (1 day) and prolonged (10 days) periods. Data are presented as the mean RFC ± SD (*n* = 5). Statistical significance is denoted by asterisks (*p < 0.05; **p < 0.01; ***p < 0.001) indicating differences between the PAT-exposed and control mice kidneys

Global DNA methylation is governed by a combination of DNMTs and MBD2. After observing PAT induced global DNA hypomethylation in kidneys, we then determined its effect on the transcript expression levels of *DNMT1, DNMT3A, DNMT3B,* and *MBD2.* We PAT significantly decreased the mRNA levels of *DNMT3A* (day 1, *p* = 0.0016; 10 days, *p* = 0.0034; Fig. 6b), and *DNMT3B* (day 1, *p* = 0.0003; 10 days, *p* = 0.0011; Fig. 6c), while significantly increasing *DNMT1* (day 1, *p* = 0.0009; 10 days, *p* = 0.0006; Fig. 6a) and *MBD2* (day 1, *p* = 0.0180; 10 days, *p* = 0.0165; Fig. 6d) in the kidneys. PAT promotes DNA hypomethylation, by upregulating the expression of the *DNMT1* and *MBD2* (coding for demthylase).

## Discussion

The global concern over food contamination by PAT has drawn attention to its potential impact on kidney health (Pal et al. 2017; Ioi et al. 2017). The kidney plays a vital role in excretion and tubular reabsorption, rendering it highly susceptible to PAT-induced injury (Ráduly et al. 2021). Renal injury occurs as a result of changes in multiple molecular pathways associated with inflammation, cell proliferation, and apoptosis (Priante et al. 2019). Recently, a surge in mycotoxin-induced kidney damage has been observed; using mice kidneys, exposed to PAT, we investigated its effect on the ERK/MAPK and PI3K/AKT pathways, as well as global DNA methylation.

Alpha-adrenergic receptors are integral components in kidney physiology, where they regulate essential functions such as renal tone, metabolism, and Na^+^ reabsorption (Pal et al. 2017). In vitro studies on PAT in kidney cell lines, demonstrated its impact on adrenergic receptor signalling that leads to severe renal toxicity (Pillay et al. 2020). Consistent with these findings, our study revealed a significant downregulation of *ADRA1A* expression by PAT in mice kidneys. The mRNA levels of *ADRA2A* also decreased significantly (acute and prolonged PAT exposure), whereas *ADRA2B* exhibited a complex response, increasing after acute exposure but decreasing after prolonged exposure. These observations align with previous work by Pillay et al. (2020), who reported an increase in the expression of *ADRA2B* following 1 day of exposure. The initial upregulation of the α_2B_-receptor observed can be attributed to the kidney’s adaptive response to counteract the effects of PAT toxicity. However, with continued PAT exposure (10 days), the response is reversed.

Hering et al. (2020) showed that the activation of α_2_ receptors can lead to vasoconstriction and reduced renal blood flow while also influencing sodium and water handling in the kidneys, ultimately affecting blood pressure regulation and overall renal function. Notably, both α_1_ and α_2_ adrenergic receptors are essential for modulating the MAPK/ERK and PI3K/AKT pathways (Karkoulias et al. 2006). The activation of α_1A_-adrenergic receptors induces the activation of ERK through an endocytic pathway (Liu et al. 2011a). Thus, when PAT suppresses the α1A-adrenergic receptors, ERK is, in turn, affected along with its associated pathways; hence disrupting the overall kidney physiological balance.

The ERK/MAPK and PI3K/AKT pathways are primarily responsible for regulating genes involved in cell proliferation, inflammation, autophagy, and apoptosis (He et al. 2021). A study by Liu et al. (2017) reported that PAT triggers the MAPK pathway in mice kidneys and increased the expression of *ERK1/2* (Liu et al. 2006), which influences cell survival and function. Additionally, Pillay et al. (2020) found that PAT altered the expression of α_1_-adrenergic receptors associated with the ERK/MAPK signalling. PAT also induced the generation of reactive oxygen species (ROS) through GSH depletion. Overproduction of ROS activates phosphorylation pathways such as MAPK and PKC (Wu et al. 2008), which may explain the increased *MAPK* mRNA expression and ERK/MAPK protein expressions by PAT. The activation of the MAPK pathway further contributes to the phosphorylation and activation of Nrf2, promoting the transcription of antioxidants (Yu et al. 2000). The imbalance between free radicals and antioxidants results in oxidative stress, which significantly contributes to the development and progression of kidney injury.

PAT triggers apoptosis by inducing a reduction in mitochondrial membrane potential, resulting in the accumulation of reactive oxygen species (ROS), while concurrently causing a decline in superoxide dismutase (SOD), GSH, and catalase (CAT) levels (Zhang et al. 2015). Glutathione is responsible for endogenous protection of the kidney against oxidative damage and serves as a regulator of redox and cell signalling pathways, including the PI3K/AKT pathway (Pillay et al. 2020; Lash 2005). The AKT pathway regulates cell-cycle progression and tumour cell growth (Porta et al. 2014); thus, its suppression could potentially lead to cell death and apoptosis (Pillay et al. 2020). In this study we observed a significant decrease in *PI3K* and increase in *AKT* mRNA expression in all mice kidneys exposed to PAT. Notably, previous studies suggested that the activation of *PI3K* leads to the phosphorylation and activation of *AKT* (Chen et al. 2020). Therefore, suppression in the expression of *PI3K* results in the suppression of *AKT* expression, and considering the importance of AKT in homeostasis, its inhibition may negatively affect the kidney (Franke et al. 2003).

Alpha-1 adrenergic receptors directly alter the ERK/MAPK pathway (Cotecchia 2010) and indirectly stimulate the activation of PI3K in keratinocytes (Calautti et al. 2005). The downregulation of the α_1_-adrenergic receptors gene expression and upregulation of ERK and MAPK protein expression in PAT-exposed mice kidneys, supports previous findings of a cross-talk between α_1_-adrenergic receptors and the ERK1/2/MAPK pathway (Morelli et al. 2014). The interplay between the PI3K/AKT and ERK/MAPK pathways, characterized by both positive and negative influences on each other, introduces complex dynamics potentially associated with DNA damage (Aksamitiene et al. 2012). There was a significant increase in *MAPK* and *AKT* with a concomitant decrease in *PI3K* expression in response to PAT exposure. This observation provides further evidence that the PAT-induced suppression of α_1_-adrenergic receptor pathways indirectly affects the activation of PI3K, and leads to pathogenesis of the kidney (Porta et al. 2014; Calautti et al. 2005). Moreover, given the association of the PI3K/AKT and ERK/MAPK pathways in carcinogenesis (Pillay et al. 2020), our findings suggest that PAT promotes kidney injury.

DNA methylation, is important in regulation and expression. Several studies have shown that mycotoxins can alter DNA methylation patterns (Sugiyama et al. 2021; Zhu et al. 2014; Ghazi et al. 2020), and affect gene expression. Our study substantiated these findings and showed that PAT significantly decreased *DNMT3A* and *DNMT3B* expression in the kidneys of mice. These findings are consistent with existing research suggesting that mycotoxins reduce the expression and activities of DNMTs, consequently leading to a decrease in global DNA methylation (Sugiyama et al. 2021; Chuturgoon et al. 2014). This global decrease in 5-mC content has been previously associated with renal injury and carcinogenesis (Bomsztyk and Denisenko 2013).

Moreover, another study supporting our findings indicated that in inflammatory-associated diseases, the methylation level of DNA is notably lower, and that the *DNMT1* and *MBD2* mRNA expressions were elevated in the diseased state (Liu et al. 2011b). PAT promotes oxidative stress in kidney cells (Zhang et al. 2015), which induces inflammation by activating pro-inflammatory signalling pathways and triggering the release of pro-inflammatory cytokines, thereby contributing to renal tissue damage and inflammatory responses (Mittal et al. 2014). In our study, PAT increased the mRNA levels of *MBD2* in the mice kidneys, which strengthens the premise that there is a positive correlation between *DNMT1* and *MBD2* mRNA levels. Furthermore, the upregulation of *MBD2* expression (increases expression of demethylase) results in lower levels of global DNA methylation (Liu et al. 2011b). These findings collectively highlight the significant impact of PAT on DNA methylation patterns and its potential role in kidney injury and inflammatory responses.

The suppression of the α_1A_-adrenergic receptors and upregulation of *DNMT1* expression, is suggestive of a potential link between DNA methyltransferase activity and the expression of the α_1A_-adrenergic receptors. PAT significantly reduced *ADRA1A* gene expression and increased *DNMT1* mRNA levels in mice kidneys. Our observation is supported by a study that revealed that the inhibition of DNMT1 activity was sufficient to increase α_1D_-adrenergic receptor mRNA and protein levels in DU145 human prostate cancer cells (Michelotti et al. 2007). However, further research is required to conclude that PAT-induced alterations of DNMT1 suppresses α_1A_-adrenergic receptors. This study was limited to global DNA methylation analysis only. Future studies will assess the promoter DNA methylation of α_1A_-adrenergic receptors induced by PAT over longer exposure times. Another limitation of this study is the lack of western blot analysis for DNMT1, DNMT3A, DNMT3B, MBD2, and alpha-adrenergic receptors due to sample limitation and the original ethical approval by the institution.

## Conclusion

PAT exposure (acute and chronic) in mice leads to the suppression of kidney α_1A_-adrenergic receptor expression and alters the ERK/MAPK and PI3K/AKT pathways. The disruption of the MAPK/ERK pathway contributes to kidney injury by promoting inflammation and cell death due to excessive oxidative stress. Further, we observed altered *PI3K* and *AKT* gene expression, suggesting impaired renal function, that can lead to kidney fibrosis and chronic kidney injury. Additionally, PAT induced global DNA hypomethylation, that may contribute to tumorigenesis. Future research should focus on integrating PAT toxicity and its influence on gene promoter methylation and genome stability, cell death, and mitochondrial dysfunction in the kidney.

### Supplementary Information

Below is the link to the electronic supplementary material.

Supplementary file 1 (DOCX 14 KB)

## Data Availability

All datasets generated in this study are available from the corresponding author on reasonable request.
