# Supplementary material for: Patulin alters alpha-adrenergic receptor signalling and induces epigenetic modifications in the kidneys of C57BL/6 mice
Source: Arch Toxicol. 2024 May 28;98(7):2143–52. doi: 10.1007/s00204-024-03728-z (PMC11168996; doi:10.1007/s00204-024-03728-z)
Supplement: Supplementary file 1 — (DOCX 14 KB) [file 204_2024_3728_MOESM1_ESM.docx]

**Supplementary Data**

**Table S1** Primer sequences and annealing temperatures used for qPCR

| Gene | Annealing Temperature (°C) | Primer Sequences (5’-3') |
| --- | --- | --- |
| *DNMT1* | 60 | F: AGAGACCAGGATAAGAAACGCA  R: CTCCTTTGATTTCCGCCTCAAT |
| *DNMT3A* | 62 | F: GGCCGAATTGTGTCTTGGTG  R: CCATCTCCGAACCACATGAC |
| *DNMT3B* | 60 | F: AGCGGGTATGAGGAGTGCAT  R: GGGAGCATCCTTCGTGTCTG |
| *MBD2* | 58 | F: AGAACAAGGGTAAACCAGACCT  R: ACTTCACCTTATTGCTCGGGT |
| *ADRA1* | 54.7 | F: CGGTGACTCACTACTACATTGTC  R: GACGCTGTGCAGCATAAGAC |
| *ADRA2A* | 57.3 | F: GTGACACTGACGCTGGTTTG  R: CCAGTAACCCATAACCTCGTTG |
| *ADRA2B* | 54.1 | F: TCTTCACCATTTTCGGCAATGC  R: AGAGTAGCCACTAGGATGTCG |
| *MAPK* | 58.2 | F: GGTTGTTCCCAAATGCTGACT  R: CAACTTCAATCCTCTTGTGAGGG |
| *MAPK14* | 55.5 | F: TGACCCTTATGACCAGTCCTTT  R: GTCAGGCTCTTCCACTCATCTAT |
| *PI3K* | 56.5 | F: CTCTCCTGTGCTGGCTACTGT  R: GCTCTCGGTTGATTCCAAACT |
| *AKT* | 55.2 | F: CTTCCGTCCACTCTTCTCTTTC  R: ATCCCCTCAACAACTTCTCAGT |
| *GAPDH* | Housekeeping | F: AGGTCGGTGTGAACGGATTTG  R: TGTAGACCATGTAGTTGAGGTCA |

**Quantification of global DNA methylation formulae:**

$$5-Methylcytosine \left( \mathrm{ng} \right)= \frac{Sample OD-Negative Control OD}{Input DNA (Slope\times2)}\times100\%$$

$$5-Methylcytosine \left( \% \right)= \frac{5-Methylcytosine (ng)}{Input DNA (ng)}\times100\%$$
